# Multidetector computed tomography of mesenteric ischaemia

**DOI:** 10.1007/s13244-014-0361-1

**Published:** 2014-10-31

**Authors:** Andreu F. Costa, Vijay Chidambaram, Jonghun J. Lee, John Asquith, Elie R. Skaff, Seng Thipphavong

**Affiliations:** 1 Joint Department of Medical Imaging, University Health Network, Mount Sinai Hospital, Women’s College Hospital, 76 Grenville St, Rm 2238, 2nd Floor, Toronto, ON M5B 1S2 Canada; 2 University of Toronto, Toronto, ON Canada; 3 Department of Radiology, The Royal Liverpool University Hospital, Liverpool, UK; 4 Department of Radiology, University Hospital of North Staffordshire, Stoke-on-Trent, UK; 5 Department of Medicine, University of Western Ontario, London, ON Canada

**Keywords:** Mesenteric ischaemia, Acute mesenteric ischaemia, Chronic mesenteric ischaemia, Ischaemic colitis, Multidetector computed tomography

## Abstract

Mesenteric ischaemia comprises a broad, heterogeneous group of diseases characterised by inadequate blood supply to the small or large bowel. Acute mesenteric ischaemia is a surgical emergency, with significant associated morbidity and mortality. Because the clinical presentation of mesenteric ischaemia is variable and often nonspecific, a high index of clinical and radiologic suspicion is required for early diagnosis. The severity of mesenteric ischaemia ranges from transient, localised ischaemia to frank necrosis of the bowel. The most common causes of acute mesenteric ischaemia are embolic and thrombotic occlusion of the superior mesenteric artery, whereas chronic mesenteric ischaemia is almost always associated with generalised atherosclerotic disease. Multidetector computed tomography (MDCT) angiography is the preferred imaging test for acute and chronic mesenteric ischaemia. MDCT is useful in making a prompt, more precise diagnosis of mesenteric ischaemia, as well as identifying the cause and potential complications, which are key to reducing patient morbidity and mortality. In this article, we review the clinical features and aetiologies of mesenteric ischaemia and illustrate the imaging manifestations on MDCT.

• *Acute and chronic mesenteric ischaemia are morbid conditions challenging to diagnose.*

• *MDCT is the first-line imaging test for evaluating patients with suspected mesenteric ischaemia.*

• *Bowel findings include wall thickening, abnormal enhancement, pneumatosis and luminal dilation.*

• *Vascular occlusion, portomesenteric venous gas, mesenteric congestion and free air can be seen.*

## Introduction

Mesenteric ischaemia comprises a complex, heterogeneous group of disorders that result in inadequate blood supply to the small or large bowel. In the acute setting, mesenteric ischaemia is a surgical emergency, with mortality rates ranging between 30 and 90 % [1]. Because the clinical presentation and imaging manifestations of mesenteric ischaemia are variable and often nonspecific, a high index of clinical and radiologic suspicion is required for prompt diagnosis and treatment [2]. The severity of mesenteric ischaemia ranges from localised, transient ischaemia to frank necrosis of the gastrointestinal tract and is a function of multiple factors, including the degree of vascular compromise, duration of the insult, metabolic requirements of the affected bowel and capacity of the underlying systemic circulation, including collateral flow [2]. In this article, we provide an overview of the epidemiology, aetiology, clinical presentation and imaging manifestations of acute and chronic mesenteric ischaemia.

## Epidemiology

Bowel ischaemia accounts for approximately 0.1 % of all hospital admissions and 1.0 % of admissions for an acute abdomen [1, 2]. Mesenteric ischaemia predominantly affects the elderly, particularly those with comorbid conditions such as congestive heart failure, cardiac arrhythmias, valvular heart disease, coronary artery disease, peripheral vascular disease, dyslipida, recent myocardial infarction or hypotensive episode [2, 3]. Tobacco use is a strongly associated risk factor [2]. In chronic mesenteric ischaemia, women are affected more often than men by a factor of 3–4:1 [2, 3], and there is invariably a background of atherosclerotic disease and often smoking. Younger patients who develop acute or chronic mesenteric ischaemia usually have a predisposing condition such as vasculitis, collagen vascular disease, hypercoagulable state or vasoactive medications [3].

## Clinical features

The clinical and laboratory features of acute mesenteric ischaemia (AMI) are nonspecific and make early diagnosis a challenge. Patients present with vague symptoms, such as abdominal pain, nausea, vomiting, diarrhoea and bloating [1, 2]. The physical examination is often benign, with an initially soft and nontender abdomen; the classic hallmark is pain out of proportion to the physical examination [1, 3]. Laboratory tests are neither sensitive nor specific; leukocytosis with leftward shift, acidosis with a high anion gap and elevated amylase may occur late [3, 4]. Though suggestive of ischaemia, an elevated lactate value is also nonspecific and a late marker [1, 2]. Stools may contain occult blood and potentially fatal haemorrhage can occur with bowel infarction [3].

Patients with chronic mesenteric ischaemia have a more insidious onset of disease, with subtle or nonspecific symptoms, an unremarkable physical examination and nonspecific laboratory tests. The classic clinical triad of postprandial abdominal pain, sitophobia (aversion to food) and weight loss may be present [5]. There may also be a history of nausea, vomiting, diarrhoea and signs of malabsorption [6].

## Aetiology

A detailed discussion of the complex pathophysiology regarding mesenteric ischaemia is outside the scope of this article, but available elsewhere [2, 7, 8]. Mesenteric ischaemia presents acutely in 95 % of cases and can be arterial or venous in origin according to the following four categories [2]: embolic occlusion of the superior mesenteric artery (SMA), 40–50 % of cases; acute mesenteric arterial thrombosis, 20–30 %; nonocclusive mesenteric ischaemia (NOMI), 25 %; mesenteric venous thrombosis (MVT), 5–15 % [6, 9].

Mesenteric emboli usually originate from left-sided cardiac chambers or valves [2, 3]. Emboli most commonly affect the SMA because of its high flow rate and acute angle with the aorta [2, 4], and they typically lodge distal to the origin of the middle colic artery, resulting in sparing of the duodenum and transverse colon [1]. Twenty per cent of patients may have synchronous emboli to other viscera, such as the spleen or kidneys [2].

Acute mesenteric arterial thrombosis carries the worst prognosis and typically occurs at or near the ostia of the mesenteric arteries. There is usually a background of generalised atherosclerosis and possibly chronic mesenteric ischaemia [1, 2, 10]. NOMI results from low cardiac output and subsequent splanchnic vasoconstriction in such settings as myocardial infarction, congestive heart failure, arrhythmias, shock, sepsis, hypovolaemia and certain drugs [2, 3, 10]. NOMI is associated with a high mortality rate and is often underdiagnosed. Treatment includes administration of vasodilating agents.

MVT involves the SMV in 95 % of cases. MVT may be primary (idiopathic), but is more often secondary to predisposing hypercoagulable conditions such as portal hypertension, trauma, inflammatory or neoplastic processes, and bowel obstruction [3, 9]. With venous thrombosis, bowel ischaemia may be acute, subacute or chronic, with the clinical presentation varying from relatively asymptomatic to acutely ill patients [2]. Thrombosis of small veins draining close to the bowel are more likely to cause bowel infarction [9].

Chronic mesenteric ischaemia (CMI) accounts for 5 % of all mesenteric ischaemia cases and is almost always secondary to severe atherosclerotic disease. Less common, nonatherosclerotic causes of CMI include fibromuscular dysplasia, median arcuate ligament syndrome, radiation-induced injury, tumours encasing or obstructing major vessels, arterial dissection, Takayasu’s arteritis and other vasculitides [3, 5, 11]. As atherosclerotic disease progresses slowly over time, collateral vessels develop throughout the splanchnic circulation. Because of these collateral vessels, intestinal infarction is rare in CMI [3], and typically two of the three main vessels must be affected for symptoms to occur [6].

## Mesenteric anatomy

**Fig. 1:**
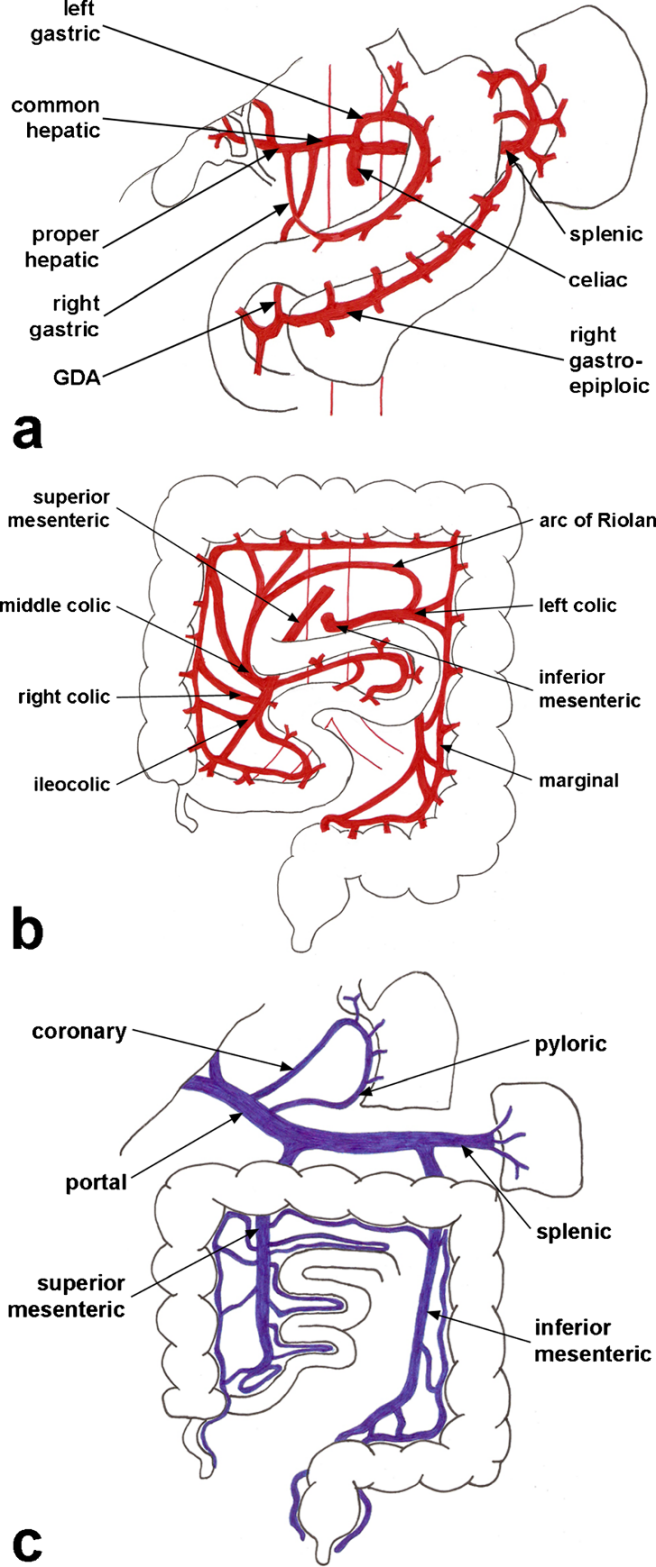
Mesenteric vasculature. Mesenteric arterial supply from the **a** coeliac and **b** superior and inferior mesenteric arteries. **c** Mesenteric venous drainage. GDA, gastroduodenal artery

Three major arteries arise from the ventral abdominal aorta to supply the splanchnic organs: the coeliac artery (CA), superior mesenteric artery (SMA) and inferior mesenteric artery (IMA). The CA (Fig. 1a) arises from the aorta at T12 or L1 and typically divides into three branches: the common hepatic artery, splenic artery and left gastric artery [2, 4]. CA branches supply the liver, distal oesophagus, stomach, pancreas, spleen, stomach and proximal duodenum.

The SMA (Fig. 1b) arises from the aorta at the level of L1 and courses inferiorly to the right lower quadrant where it terminates as the ileocolic artery [2]. The major SMA branches are the inferior pancreaticoduodenal artery, jejunal and ileal arteries, and the right and middle colic arteries. The SMA partially supplies the pancreas and distal duodenum, as well as the entire jejunum, ileum, caecum, ascending colon and the majority of the transverse colon to the splenic flexure [4]. The SMA collateralises with the CA via the pancreaticoduodenal arteries and collateralises with the IMA via the marginal artery of Drummond. Closer to the mesenteric root, the SMA and IMA variably collateralise via the arc of Riolan, which connects the middle colic artery to a left colic artery branch.

The IMA (Fig. 1b) arises a few centimetres above the aortic bifurcation at approximately L3. The IMA branches into the left colic and sigmoid arteries, and terminates as the superior rectal branch, which collateralises with the internal iliac artery [4]. Thus, the IMA supplies the splenic flexure, descending and sigmoid colon and proximal rectum. The splenic flexure is a watershed zone between the SMA and IMA perfusion territories and is at risk for ischaemia [2].

**Fig. 2:**
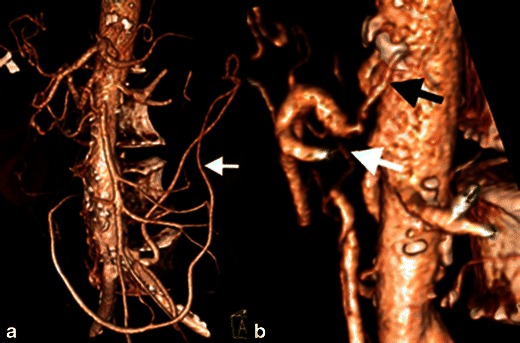
Severe chronic mesenteric ischaemia. Patient with severe, three-vessel mesenteric atherosclerosis. The coeliac artery and superior mesenteric artery are occluded. **a** The inferior mesenteric artery is severely stenosed (>75 %) and filling via a prominent marginal artery of Drummond (*white arrow*). **b** Magnified view showing severe stenosis at the origin of the coeliac (*black arrow*) and superior mesenteric (*white arrow*) arteries

In addition to the aforementioned collateral systems, severe, chronic stenosis or occlusion of all three mesenteric arteries can result in phrenic, lumbar and internal iliac collaterals [11] (Fig. 2).

Mesenteric venous blood drains via the superior and inferior mesenteric veins, which parallel the arteries, and into the portal vein (Fig. 1c). The left colic, sigmoid and superior haemorrhoidal veins drain into the inferior mesenteric vein (IMV), which joins the splenic vein. The duodenal, pancreatic, right gastroepiploic, jejunal, ileal, right and middle colic veins drain into the superior mesenteric vein (SMV).

## Overview of imaging modalities

The American College of Radiology Appropriateness Criteria advocate computed tomography angiography (CTA) as the first-line imaging modality to evaluate both acute and chronic mesenteric ischaemia [6]. CTA is fast, readily available, noninvasive and highly accurate, and it can be used to evaluate other causes of abdominal pain or provide anatomic mapping for preoperative planning [6]. CTA is also less dependent on operator and patient factors [2] than other imaging modalities.

Although previously considered the gold standard diagnostic test, and despite the added benefit of potentially guiding therapy, conventional angiography is now considered a second-line test to diagnose acute or chronic mesenteric ischaemia [6]. In the acute setting, angiography is controversial if signs of peritonitis are present and not recommended if the patient is unstable [2, 6]. The main drawbacks of angiography are that it is invasive, time consuming and not available at all medical centres, particularly after routine working hours [1].

Contrast-enhanced magnetic resonance angiography (MRA) can evaluate stenoses and occlusions of the proximal CA and SMA. However, MRA is limited in assessing smaller calibre arteries including much of the IMA and also less sensitive to detecting gas bubbles and signs of NOMI [6]. The availability and lengthy imaging times of MRA also limit its usefulness in the acute setting, where delayed diagnosis can affect patient care.

Abdominal radiographs and ultrasound (US) are of limited value, with poor sensitivity and specificity in diagnosing mesenteric ischaemia, but may be performed in patients with nonspecific abdominal pain [2, 6]. Radiographs are usually only abnormal with frank bowel infarction [6]. Although Doppler US can be used to assess for proximal mesenteric artery occlusion and signs of bowel compromise, this can be technically challenging and dependent on a number of factors, including patient body habitus, patient compliance and mobility, the presence of bowel gas and local expertise [2]. In patients with CMI, Doppler US peak systolic velocity measurements of greater than 275 cm/s in the SMA and 200 cm/s in the CA correspond to at least 70 % stenosis of these vessels. Like MRA, US cannot be used to assess for distal arterial occlusion or NOMI.

## Multidetector CT protocol

Although the majority of abdominal MDCT applications benefit from positive oral contrast with barium or iodine suspension, such intraluminal contrast makes it difficult to assess enhancement of the bowel mucosa. Evaluation of the intestinal wall is thus best performed with a neutral oral contrast agent, such as water or methylcellulose solution [3, 10, 12].

Although initial CT protocols for mesenteric ischaemia recommended an initial, unenhanced phase to assess for intramural haematoma, calcified plaque and bowel enhancement, recent articles suggest that the unenhanced phase can be omitted [9, 10, 13, 14], as a loop of normally enhancing bowel can often be found to compare and act as an internal control [13]. The multiphasic MDCT imaging protocol includes both arterial and portal venous phase acquisitions: the arterial phase is required for optimal assessment of the mesenteric arterial supply, as thromboembolic disease may account for 60–80 % of acute mesenteric ischaemia cases, and the venous phase is used for assessing bowel wall enhancement and venous drainage. Typical CT parameters are as follows: 120 ml of non-ionic iodinated contrast material is power injected at a rate of 3–5 ml/s, followed by a saline chaser; 120 kVp; 270–300 mAs with automatic tube current modulation whenever possible; as thin a collimation as possible (e.g. 0.625 mm in 64-slice scanners) because of the small size of mesenteric branches [9, 10, 12]. The arterial and portal-venous phases are acquired at approximately 30 and 60 s after injection, respectively, often triggered by a specific attenuation threshold in a region of interest placed over the abdominal aorta [13, 14].

Raw images are reconstructed into 3–5-mm-thick slices for review of the abdominal viscera. Sagittal and coronal reformatted images are generated, as well as three-dimensional maximum-intensity projection (MIP) and volume-rendered images. Sagittal reformats are particularly useful for assessing the origin of the mesenteric arteries from the aorta [10].

## MDCT imaging findings of mesenteric ischaemia

MDCT imaging features of mesenteric ischaemia can be classified as intestinal, vascular and mesenteric (Table 1) [9].

**Table 1:** Multidetector computed tomographic findings in mesenteric ischaemia (adapted from [7])

Intestinal	• Mural wall thickening
	- Hypodense oedema
	- Hyperdense haemorrhage
	- Target or halo sign
	- Bowel wall may be paper thin
	• Abnormal mural enhancement
	- Decreased enhancement
	- Increased enhancement
	- Absent enhancement
	• Bowel dilation
	• Pneumatosis intestinalis
Vascular	• Arterial embolus
	• Arterial thrombus
	• Non-occlusive mesenteric ischaemia: diminutive aorta and IVC
	• Mesenteric venous thrombosis
	- Intraluminal filling defect
	- Venous engorgement
	- Venous collaterals
	• Portomesenteric venous gas
	• Chronic mesenteric ischaemia
	- Occlusion or severe stenosis in at least two major splanchnic arteries
Mesenteric	• Mesenteric fat stranding
	• Ascites
	• Free air

### Bowel wall thickening

**Fig. 3:**
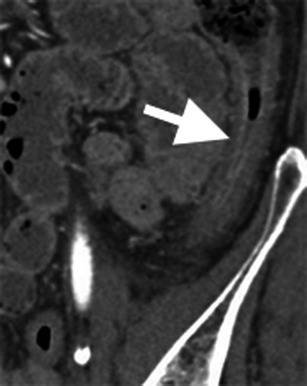
Bowel wall thickening in ischaemic colitis. Contrast-enhanced coronal reformatted image of a patient with severe chronic mesenteric ischaemia and diarrhoea. There is circumferential, oedematous wall thickening of the descending colon, pathologically proven to represent ischaemic colitis

**Fig. 4:**
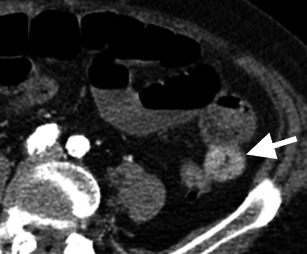
Bowel wall thickening and haemorrhage. Axial CT image in a patient with an occluded superior mesenteric artery. A short segment of jejunum shows circumferential thickening and hyperattenuation, favoured to represent bowel wall heamatoma

Bowel wall thickening is the most common intestinal CT finding in mesenteric ischaemia (Fig. 3) [3]. Mural thickening is typically circumferential and measures approximately 0.8 cm, but can reach up to 1.5 cm, particularly in the setting of venous thrombosis [10]. A “target” or “halo” sign may be present, representing a two- or three-layer striated pattern of enhancement. This sign is assumed to represent hypodense oedema or inflammation in the submucosal layer, sandwiched by enhancing mucosa and muscularis propria [3, 15]. If present, acute haemorrhage will appear as hyperattenuating material in the bowel wall (Fig. 4) [9, 10]. The absence of bowel wall thickening does not exclude mesenteric ischaemia, however. In cases of acute arterio-occlusive transmural infarction, the bowel wall can become paper thin [10].

The distribution of bowel wall thickening depends on the aetiology of ischaemia. In SMA or SMV occlusion, for example, the small bowel, right colon and proximal transverse colon are thickened [3, 10], whereas in NOMI, findings are often much more diffuse. Segments of ischaemic bowel may be multifocal with mesenteric emboli [3].

### Bowel wall enhancement

**Fig. 5:**
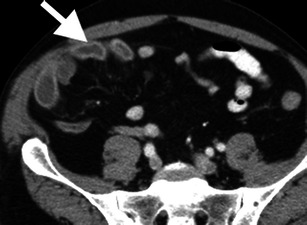
Hyper-enhancement of the bowel mucosa. Axial contrast-enhanced CT image demonstrates hyper-enhancement of the right lower quadrant ileal mucosa (*arrow*) secondary to superior mesenteric artery occlusion (not shown). The degree of mucosal enhancement can be compared to left-sided bowel loops as an internal control

Bowel wall enhancement may be increased, decreased or absent with mesenteric ischaemia (Fig. 5). Complete lack of mural enhancement is a highly specific but insensitive finding for acute mesenteric ischaemia [3, 9, 10]. Abnormal mural enhancement is often more subtle, such as delayed arterial-phase enhancement and persistent portal-venous phase enhancement [9]. Hyper-enhancement of the bowel wall may be caused by impaired venous drainage of contrast, such as in MVT or strangulated hernia, luxury reperfusion after arterial occlusion or in cases of reduced arterial perfusion and venous drainage, such as NOMI or shock bowel. In contrast to decreased or absent mural enhancement, hyperaemia is probably a good prognostic sign as it likely indicates viable bowel [11].

### Bowel wall gas

**Fig. 6:**
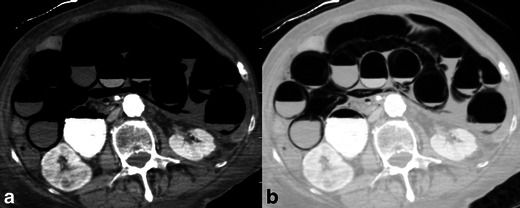
Pneumatosis intestinalis. Axial contrast-enhanced CT images in **a** soft tissue and **b** lung windows demonstrate extensive pneumatosis intestinalis secondary to superior mesenteric arterial occlusion. Note the increased conspicuity of pneumatosis with the use of lung windows

In the setting of mesenteric ischaemia, pneumatosis intestinalis (mural gas) often indicates transmural infarction, particularly if it is associated with bandlike portomesenteric venous gas (Fig. 6) [10, 16]. However, pneumatosis and venous gas may also be seen with reversible ischaemia [9, 16], as well as a plethora of non-ischaemic disorders such as chronic pulmonary disease, medications and infectious, inflammatory or neoplastic causes of intestinal mucosal disruption [3, 17]. There is in addition a primary (idiopathic), asymptomatic form termed pneumatosis cystoides intestinalis, which is characterised by circular, bubble-like gas collections in the bowel wall and mesentery; it almost always affects the colon [17].

### Bowel lumen

**Fig. 7:**
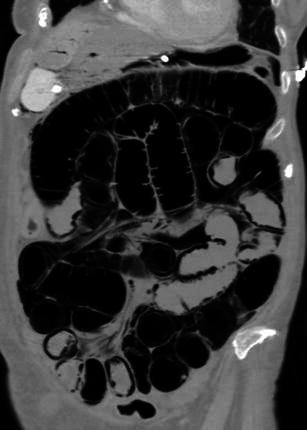
Dilated bowel in acute ischaemia. Coronal reformatted CT image demonstrates diffuse small bowel dilation with pneumatosis in a patient with extensive small bowel ischaemia. Portal venous gas in the liver is also evident

Bowel dilation (Fig. 7) is often present and secondary to either aperistalsis from ischaemic injury or complete loss of contractility from transmural infarction [18]. Severe dilation is seen in the setting of irreversible, transmural ischaemia or infarction [10].

### Mesenteric arteries

**Fig. 8:**
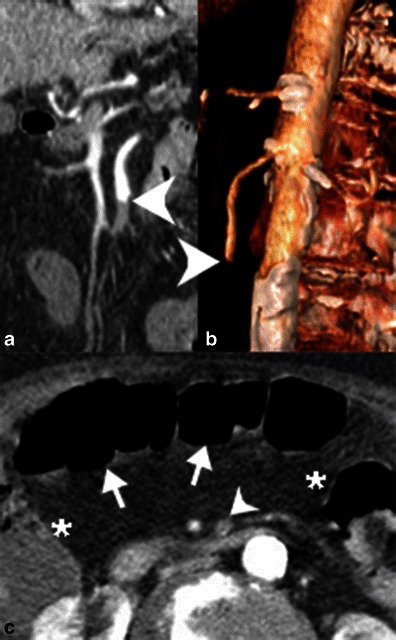
Embolic occlusion of the superior mesenteric artery. **a** Contrast-enhanced coronal reformatted CT image and **b** 3D volume-rendered reconstruction of demonstrate an abrupt cutoff of the proximal superior mesenteric artery (*white arrowheads*) secondary to an embolus. **c** Axial contrast-enhanced CT demonstrates relatively poor enhancement of the transverse colon (*white arrows*) relative to the hepatic and splenic flexures (*). Note also the occluded SMA (*white arrowhead*)

**Fig. 9:**
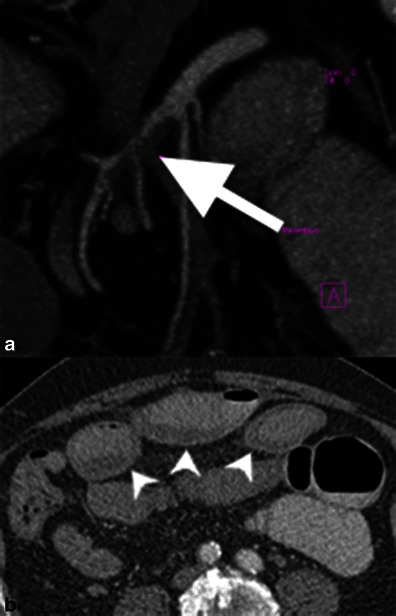
SMA thrombosis. **a** Contrast-enhanced, coronal reformatted CT images demonstrate an eccentric filling defect in the proximal SMA, corresponding to thrombus formation. **b** Axial contrast-enhanced CT image shows oedematous bowel wall thickening of small bowel loops (*white arrowheads*) and mesenteric congestion. This patient subsequently improved with conservative anticoagulation therapy

Emboli typically lodge in the proximal SMA, distal to the origin of the middle colic artery. SMA embolism appears as a centrallylocated, hypodense intraluminal filling defect (Fig. 8). In contrast, SMA thrombosis typically develops eccentrically at or near the ostium, on a background of atherosclerosis (Fig. 9).

**Fig. 10:**
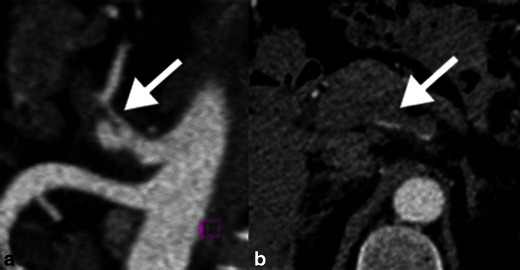
Spontaneous coeliac artery dissection. **a** Contrast-enhanced sagittal reformatted image demonstrates a dissection flap of the coeliac artery (*white arrow*). **b** Axial CT image shows the dissection flap continues into the common hepatic artery with thrombosis of the false lumen. There was no evidence of bowel ischaemia in this patient and the appearance remained stable on follow-up imaging

**Fig. 11:**
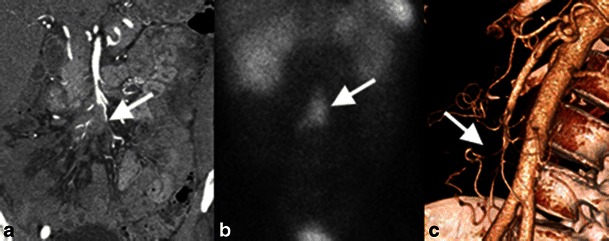
Mesenteric ischaemia secondary to vascular compression by a small bowel carcinoid. **a** Coronal contrast-enhanced reformatted image shows mesenteric metastatic disease from the small bowel carcinoid encasing distal branches of the SMA (*white arrow*). **b** Coronal image from an octreotide scan shows uptake by the metastatic disease (*white arrow*), indicating tumoral somatostatin receptors. **c** Three-dimensional volume-rendered reconstruction shows attenuated calibre of the ileocolic artery (*white arrow*)

Another potential, albeit less common cause of AMI is dissection of a splanchnic artery, often as a continuation of aortic dissection. The most common CT findings include an intimal flap, aneurysmal dilation and a thrombosed false lumen (Fig. 10) [19]. In dissections causing subtotal luminal occlusion, the branching smaller arteries are at higher risk of being affected. Malignant encasement and narrowing of the mesenteric arteries is another uncommon cause of AMI (Fig. 11).

### Portomesenteric veins

**Fig. 12:**
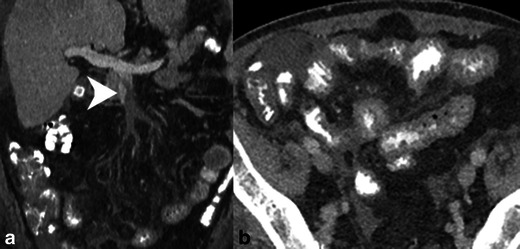
SMV thrombosis. **a** Contrast-enhanced coronal reformatted CT demonstrates occlusive thrombus in the SMV near the portal confluence (*white arrowhead*). **b** Axial CT image demonstrates diffuse small bowel wall thickening secondary to venous congestion and ischaemia

**Fig. 13:**
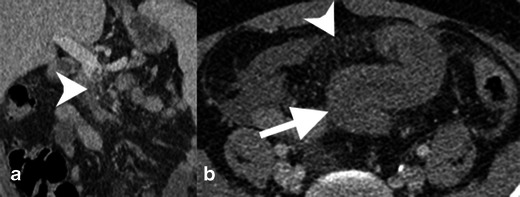
SMV thrombosis with mesenteric congestion. **a** Contrast-enhanced coronal reformatted CT of an occlusive thrombus in the SMV (*white arrowhead*) extending to the portal confluence. **b** Axial contrast-enhanced CT image demonstrates mesenteric congestion with engorged mesenteric veins and trace fluid (*white arrowhead*). The small bowel is thickened because of the venous stasis and probable ischaemia

**Fig. 14:**
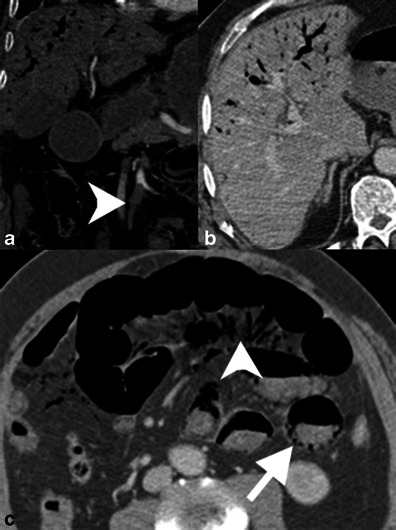
Portomesenteric venous gas. **a** Contrast-enhanced coronal reformatted CT demonstrates occlusion of the SMA (*arrowhead*). Portal venous gas is seen in the liver. **b** Axial CT shows portal venous gas in the anti-dependent liver. **c** The small bowel demonstrates luminal dilation, a paper-thin wall and poorly enhancing mucosa. There is mesenteric venous gas (*arrowhead*) as well as pneumatosis in the dependent bowel wall. The patient had established bowel necrosis and died shortly after imaging

SMV thrombosis is demonstrated as a partial or complete hypoattenuating filling defect (Figs. 11 and 12) [9]. Care must be taken to avoid mistaking delayed venous filling and other flow-related phenomena for thrombi. The affected venous branch may be enlarged, particularly if the thrombus is acute or malignant tumour thrombus. The associated venous congestion can result in engorged mesenteric veins (Fig. 12) [9]. As mentioned previously, in the setting of ischaemia portomesenteric venous gas and bandlike pneumatosis are highly associated with transmural bowel infarction (Figs. 13 and 14) [16].

MVT secondary to malignancy typically occurs with hepatocellular carcinoma and pancreatic adenocarcinoma, but can be seen with other malignancies as well [9].

### Non-occlusive mesenteric ischaemia

Because it is a systemic disorder, MDCT findings of NOMI are typically diffusely thickened and fluid-distended bowel loops and markedly attenuated, pruned blood vessels [10].

**Fig. 15:**
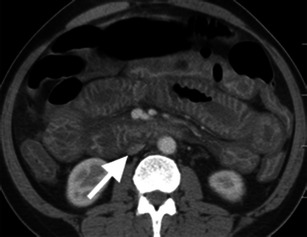
Shock bowel. Contrast-enhanced axial CT image demonstrates diffuse bowel wall thickening with mural hyper-enhancement. Note the secondary sign of a flattened inferior vena cava, indicating hypovolaemia

Shock bowel is a subtype of NOMI (Fig. 15). Typical MDCT features of shock bowel include diffuse small bowel thickening with relative sparing of the colon, prolonged mural enhancement, fluid or gas-filled small bowel loops, and other signs of hypovolaemia, such as a flattened inferior vena cava (IVC) [3].

### Chronic mesenteric ischaemia

The diagnosis of CMI is based on clinical symptoms and supported by imaging findings, following exclusion of other potential intestinal disorders [3]. CT accurately demonstrates calcified and noncalcified plaque causing arterial stenosis or occlusion, typically in the proximal CA and SMA [10]. Small, attenuated vessels and large collateral vessels are important supportive findings (Fig. 2) [10].

### Mesenteric stranding, fluid and gas

With the exception of free peritoneal gas, mesenteric MDCT findings in bowel ischaemia are nonspecific, as they are commonly seen in any acute abdominal process [3]. Ascites and stranding or haziness of the mesenteric fat are seen to variable degrees, and, as these processes often result from venous congestion, are much more common in MVT than arterio-occlusive disorders [9].

In contrast to free fluid and fat stranding, free air in the setting of mesenteric ischaemia is indicative of bowel infarction and perforation.

## Conclusion

Acute and chronic mesenteric ischaemia are morbid conditions that are challenging to diagnose. Patients present with variable, nonspecific signs and symptoms, and the physical examination is often benign. A high index of clinical and radiologic suspicion is thus required for diagnosis. Contrast-enhanced, multidetector CT angiography is the first-line imaging test for evaluating patients with suspected acute or chronic mesenteric ischaemia. MDCT imaging features can be categorised according to bowel, vascular and mesenteric findings, and they include bowel wall thickening, altered enhancement, luminal dilation, arterial thromboembolic disease, venous thrombosis, mesenteric fluid and fat stranding. In the right clinical setting, pneumatosis, portomesenteric venous gas and free air are highly specific but insensitive signs of bowel infarction. Familiarity with the MDCT imaging manifestations of mesenteric ischaemia allows for a more precise, prompt diagnosis, early institution of therapy and potentially improved patient outcomes.
